# Supplementary material for: Provision of medical same day emergency care services within the UK: analysis from the Society for Acute Medicine Benchmarking Audit
Source: BMJ Open. 2025 Apr 22;15(4):e094580. doi: 10.1136/bmjopen-2024-094580 (PMC12015722; doi:10.1136/bmjopen-2024-094580)
Supplement: online supplemental file 1 [file bmjopen-15-4-s001.docx]

**Supplementary Table 1: Hospital sites included in patient-level and organisational-level SDEC analysis.**

|  | Overall | England | Scotland | Wales | Northern Ireland |
| --- | --- | --- | --- | --- | --- |
| Estimated eligible units* | 247 | 190 | 30 | 15 | 12 |
| Patient level data (unplanned attendances) | 188 (76.1%) | 159 (83.7%) | 12 (40%) | 10 (66.6%) | 7 (58.3%) |
| Organisational survey | 140 (56.6%) | 122 (64.2%) | 8 (26.7%) | 6 (40%) | 4 (33.3%) |

**Supplementary Table 2:** Compliance with standards for SDEC, with comparison by hospital size. SOP: Standard Operating Procedure. *Chi square test comparing proportion with vs without SOP.

|  | **Overall**  (N = 139) | **Smaller Hospitals**  (N = 40) | **Medium Hospitals**  (N = 47) | **Larger Hospitals**  (N = 52) | **P value** |
| --- | --- | --- | --- | --- | --- |
|  | *n (%)* | *n (%)* | *n (%)* | *n (%)* |  |
| **Consultant physically available** | 89 (64) | 30 (75) | 29 (62) | 30 (58) | 0.21 |
| **Nominated clinician for overall leadership** | 118 (85) | 32 (80) | 40 (85) | 46 (88) | 0.53 |
| **Contact non-attenders** | 110 (80) | 28 (72) | 37 (79) | 45 (87) | 0.22 |
| **Collect patient feedback** | 114 (82) | 29 (73) | 43 (91) | 42 (81) | 0.07 |
| **Private area available** | 126 (91) | 36 (90) | 44 (94) | 46 (88) | 0.67 |
| **Presence of a SOP**  ***Yes***  ***No***  ***Unsure*** | 113 (81)  17 (12)  9 (6) | 31 (78)  8 (20)  1 (2) | 38 (81)  5 (11)  4 (9) | 44 (85)  4 (8)  4 (8) | 0.68* |


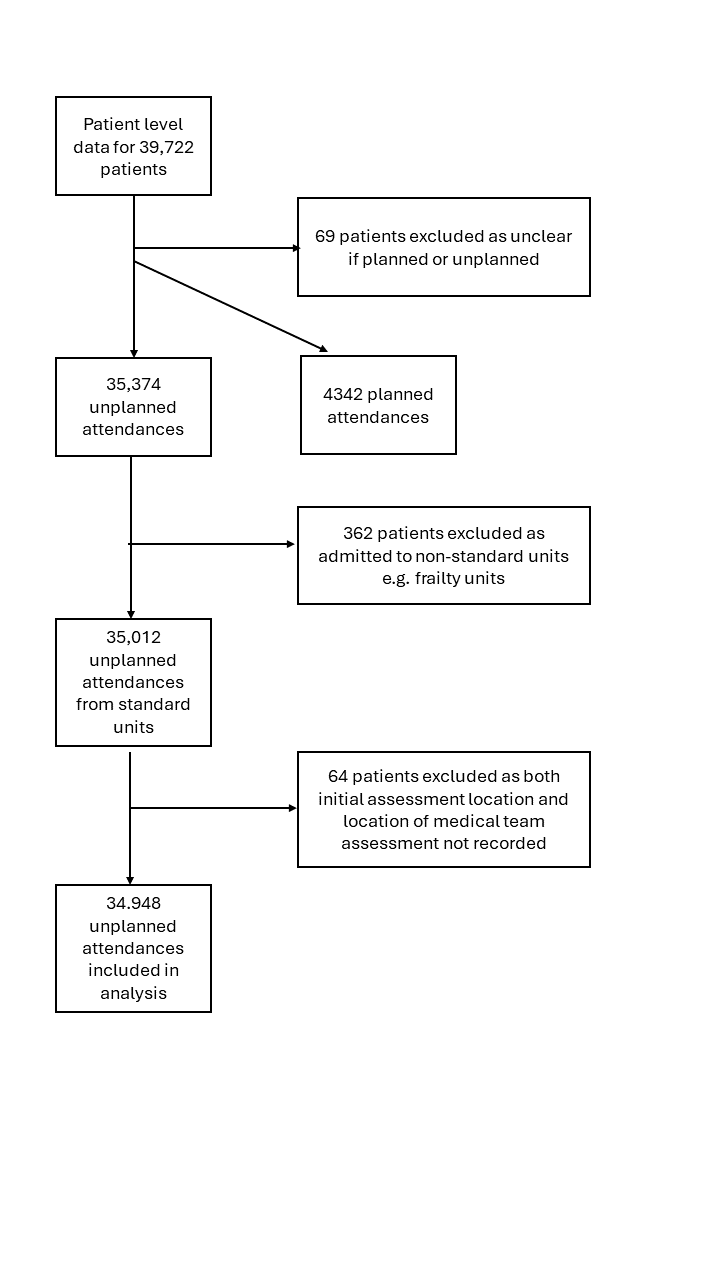


**Supplementary Figure 1:** Flow diagram showing process of exclusion for analysis of patient-level data.

**Supplementary Table 3:** Location of first assessment by the medical team, for unplanned medical attendances by year. SDEC: Same Day Emergency Care; ED: Emergency Department; AMU: Acute Medical Unit.

|  | **SDEC** | | **ED** | | **AMU** | | **Other** | | **Total** |
| --- | --- | --- | --- | --- | --- | --- | --- | --- | --- |
|  | % | *N* | % | *N* | % | *N* | % | *N* | *N* |
| **2019** | 22.1% | *1426* | 37.7% | *2431* | 36.6% | *2362* | 3.6% | *233* | *6452* |
| **2020*** | 21.6% | *1116* | 44.0% | *2274* | 31.1% | *1606* | 3.3% | *169* | *5165* |
| **2021** | 22.2% | *1797* | 40.8% | *3301* | 33.2% | *2684* | 3.7% | *301* | *8083* |
| **2022** | 24.1% | *1724* | 52.2% | *3730* | 21.7% | *1551* | 1.9% | *138* | *7143* |
| **2023** | 29.8% | *2403* | 48.5% | *3903* | 19.2% | *1542* | 2.6% | *206* | *8054* |

|  | **SAMBA19** | | **Winter SAMBA20** | | **SAMBA21** | | **SAMBA22** | | **SAMBA23** | |  |
| --- | --- | --- | --- | --- | --- | --- | --- | --- | --- | --- | --- |
| **Patient population** | *N* | *%* | *N* | *%* | *N* | *%* | *N* | *%* | *N* | *%* | *P value* |
| Age (years) *16-29*  *30-39*  *40-49*  *50-59*  *60-69*  *70-79*  *80-89*  *90+*  >70 years | 157  155  186  257  224  155  160  32  447 | 11.1%  10.9%  12.0%  18.0%  15.7%  17.9%  11.2%  2.2%  31.4% | 124  139  163  178  187  172  132  21  325 | 11.1%  12.5%  14.6%  16.0%  16.8%  15.45  11.8%  1.9%  29.1% | 247  244  273  275  256  287  178  37  502 | 13.8%  13.6%  15.2%  15.3%  14.3%  16.0%  9.9%  2.1%  27.9% | 192  204  229  296  282  288  199  34  521 | 11.1%  11.8%  13.3%  17.2%  16.4%  16.7%  11.5%  2.0%  30.2% | 295  321  299  369  269  412  282  56  750 | 12.3%  13.4%  12.4%  15.4%  15.4%  17.2%  11.8%  2.3%  31.2% | 0.002  0.137 |
| Gender *Female* | 826 | 57.9% | 671 | 60.1% | 1026 | 57.1% | 979 | 56.8% | 1358 | 56.5% | 0.267 |
| Care home resident  *(missing=9)* | 17 | 1.2% | 11 | 1.0% | 18 | 1.0% | 12 | 0.7% | 23 | 1.0% | 0.714 |
| Discharged from hospital in preceding 30 days *(missing:9)* | 159 | 11.2% | 141 | 12.7% | 237 | 13.2% | 229 | 13.3% | 366 | 15.2% | 0.008 |
| Arrival to hospital*  *00:00-08:00*  *08:00-20:00*  *20:00-23:59* | 39  1366  21 | 2.7%  95.8%  1.5% | 36  1058  22 | 3.2%  94.8%  2.0% | 62  1715  20 | 3.5%  95.4%  1.1% | 100  1586  38 | 5.8%  92.0%  2.2% | 140  2222  41 | 5.8%  92.5%  2.2% | 0.49 |
| NEWS*  0  1  2  3  4  5  6  7+  *Missing*  NEWS ≥3 | 858  327  113  58  25  9  12  6  18  110 | 60.9%  23.2%  8.0%  4.1%  1.8%  0.6%  0.9%  0.4%  7.8% | 594  287  120  64  22  14  4  4  7  108 | 53.6%  25.9%  10.8%  5.8%  2.0%  1.3%  0.4%  0.4%  9.7% | 1052  478  157  60  28  13  7  2  0  110 | 58.5%  26.6%  8.7%  3.3%  1.6%  0.7%  0.4%  0.1%  6.1% | 990  435  167  76  28  15  5  4  4  128 | 57.6%  25.3%  9.7%  4.4%  1.6%  0.9%  0.3%  0.2%  7.4% | 1133  584  230  100  44  10  8  4  290  126 | 53.6%  27.6%  10.9%  4.7%  2.1%  0.5%  0.4%  0.2%  7.9% | <0.005  0.01^ |
| CFS (in over 70s) (n=1720)  1  2  3  4  5  6  7  8  9  CFS ≥5 | (n/a) | (n/a) | (325)  45  56  84  55  34  24  21  5  1  85 | 13.9%  17.2%  25.9%  16.9%  10.5%  7.4%  6.5%  1.5%  0.3%  26.2% | (409)  39  68  105  92  42  44  13  2  4  105 | 9.5%  16.6%  25.7%  22.5%  10.3%  10.8%  3.2%  0.5%  1.0%  25.7% | (369)  44  71  119  62  43  19  9  2  73 | 11.9%  19.2%  32.3%  16.8%  11.7%  5.2%  2.4%  0.5%  19.8% | (617)  38  88  170  140  87  66  23  4  1  181 | 6.2%  14.3%  27.6%  22.7%  14.1%  10.7%  3.7%  0.7%  0.2%  29.3% | 0.001  0.012 |

**Supplementary Table 4**: Patient characteristics for unplanned attendances receiving their medical team assessment within SDEC services, compared by year. Data for 8466 unplanned attendances.

|  | **SDEC** | | **ED** | | **AMU** | | **Other** | |
| --- | --- | --- | --- | --- | --- | --- | --- | --- |
|  | % | *n* | % | *n* | % | *n* | % | *n* |
| Discharged without overnight admission | 82.4% | *6908* | 12.8% | *1980* | 15.2% | *1469* | 21.3% | *223* |
| Discharged on day 1-7 | 11.3% | *944* | 49.5% | *7676* | 52.2% | *5053* | 43.6% | *456* |
| In-hospital at day 8 - continuous stay | 4.4% | *366* | 29.5% | *4578* | 26.5% | *2572* | 27.2% | *284* |
| In-hospital at day 8 - readmitted after discharge | 0.6% | *51* | 0.9% | *133* | 0.8% | *77* | 1.1% | *11* |
| Transferred to other healthcare facility | 0.3% | *27* | 1.9% | *298* | 1.7% | *161* | 1.5% | *16* |
| Died in hospital | 0.1% | *6* | 3.6% | *558* | 2.4% | *229* | 1.8% | *19* |
| Self-discharged | 0.9% | *78* | 1.8% | *282* | 1.3% | *129* | 3.5% | *37* |

**Supplementary Table 5:** Outcomes after 7 days for unplanned attendances, by location of assessment by the medical team. SDEC: Same Day Emergency Care; ED: Emergency Department, AMU: acute medical unit.

|  | OR | P value | 95% CI |
| --- | --- | --- | --- |
| Age  16-29  30-39  40-49  50-59  60-69  70-79  80-89  90+ | *Reference*  0.89  0.81  0.62  0.47  0.33  0.22  0.17 | 0.065  <0.005  <0.005  <0.005  <0.005  <0.005  <0.005 | 0.79-1.01  0.72-0.90  0.55-0.69  0.42-0.52  0.30-0.37  0.20-0.24  0.15-0.20 |
| Gender  Female  Male  Other | *Reference*  0.87  0.78 | <0.005  0.745 | 0.82-0.92  0.18-3.45 |
| Care home residence  No  Yes | *Reference*  0.56 | <0.005 | 0.48-0.66 |
| Recent discharge  No  Yes | *Reference*  0.68 | <0.005 | 0.63-0.73 |
| NEWS score | 0.69 | <0.005 | 0.68-0.70 |
| Time of arrival  00:00-07:59  08:00-19:59  20:00-23:59 | 1.03  *Reference*  0.25 | 0.502  <0.005 | 0.95-1.11  0.22-0.28 |
| Source of referral  ED  111  GP  Other hospital  Own hospital (OPD)  Own hospital (other)  Paramedic | *Reference*  1.07  2.88  0.97  1.96  2.26  1.10 | 0.688  <0.005  0.793  <0.005  <0.005  0.238 | 0.76-1.51  2.71-3.06  0.75-1.24  1.67-2.29  1.91-2.68  0.94-1.30 |
| Year  2019  2020  2021  2022  2023 | *Reference*  1.08  1.24  1.12  1.31 | 0.088  <0.005  0.007  <0.005 | 0.99-1.19  1.14-1.35  1.03-1.22  1.21-1.43 |

**Supplementary Table 6:** Logistic regression model for likelihood of same day discharge. Pseudo R2=0.1949. OR: odds ratio; NEWS: National Early Warning Score; ED: Emergency Department; GP: general practice; OPD: outpatient department.

| **Row Labels** | **SAMBA19** | **Winter SAMBA20** | **SAMBA21** | **SAMBA22** | **SAMBA23** |
| --- | --- | --- | --- | --- | --- |
| Discharged without overnight admission | 80.1% | 81.8% | 85.3%* | 81.4% | 82.8% |
| Discharged on day 1-7 | 12.8% | 13.3% | 8.7% | 11.5% | 11.2% |
| In-hospital at day 8 - continuous stay | 4.5% | 3.5% | 4.1% | 5.2% | 4.4% |
| In-hospital at day 8 - readmitted after discharge | 1.1% | 0.7% | 0.6% | 0.5% | 0.4% |
| Transferred to other healthcare facility | 0.3% | 0.2% | 0.3% | 0.5% | 0.3% |
| Died in hospital | 0.07% | 0.09% | 0.11% | 0.06% | 0.04% |
| Self-discharged | 1.2% | 0.5% | 1.0% | 0.9% | 0.9% |

**Supplementary Table 7:** Outcomes at day 8 for unplanned attendances receiving medical assessment in SDEC, by year.
