# Supplementary material for: Provision of medical same day emergency care services within the UK: analysis from the Society for Acute Medicine Benchmarking Audit
Source: BMJ Open. 2025 Apr 22;15(4):e094580. doi: 10.1136/bmjopen-2024-094580 (PMC12015722; doi:10.1136/bmjopen-2024-094580)
Supplement: online supplemental appendix 1 [file bmjopen-15-4-s002.docx]

**Appendix 1: SAMBA22 questions regarding Medical Same Day Emergency Care**

(or equivalent – names may differ, but include Same Day Emergency Care (SDEC), Ambulatory Emergency Care (AEC), and Rapid Assessment and Care (RAC), providing assessment and treatment for medical patients with zero-length of stay)

1. Do you have a medical SDEC service (or equivalent)? Y/N
2. If yes: What is it called? ……………………….
3. Is your SDEC provided in a unit that is physically separate to the AMU? Y/N
4. Does your SDEC only see patients attending with specific conditions (e.g. DVT) or on protocolised pathways? Yes/no
   1. If yes: Can patients presenting to ED with these conditions be directed to SDEC without clerking by an ED clinician? Yes/no
5. From what time are patients seen in your SDEC service? Time/24hrs
6. Until what time are patients in your SDEC service? Time/24hrs
7. Is a consultant physician physically available throughout the opening times of your SDEC? Y/N
8. Do you have a nominated clinician for overall leadership of the SDEC service? Y/N
   1. If Y: from which staff group? Consultant/nursing/ACP/other…………
9. Do you contact non-attenders? Y/N
10. Do you book patients to return to SDEC? Y/N
    1. If Y: Are they booked to a time slot? Y/N
    2. If Y: What can patients return for: select all that apply: acute medicine review/IV antibiotics/CT scans/US scans/anaemia treatment/DVT rule out/specialty review
11. Do you collect patient feedback? Y/N
12. Do you have a SOP (standard operating procedure) for the SDEC? Y/N/unknown
13. Do you have specific SDEC/ambulatory pathways for: (tick all that apply)

Chest pain/PE/DVT/anaemia/AF/AKI/deranged liver function tests /headache/papilloedema/cellulitis/heart failure/Covid/None

1. Do you have SDEC specific patient information available? Y/N
2. Is there a private area available and accessible within SDEC (e.g. for confidential conversation)? Y/N
3. How many assessment spaces (for patient review) are in your SDEC? (number)
4. Does your SDEC accept patients requiring assistance with mobility? Y/N
5. Does your SDEC accept patients confined to:
   1. Bed Y/N
   2. Chair Y/N
6. Do you use set criteria to assess patient suitability for SDEC? Y/N
   1. If Y: Does this include-
      1. Amb score Y/N
      2. GAPS Y/N
      3. CFS Y/N
      4. NEWS2 (or equivalent) Y/N
      5. Centre specific criteria Y/N
7. Can ED refer patients to SDEC from ED triage (without full clinician review)? Y/N
8. Can ED refer patients to SDEC without prior discussion with the medical team? Y/N
9. Can GP/primary care refer patients directly to SDEC for first medical assessment? Y/N
10. Can paramedics refer patients directly to SDEC for first medical assessment? Y/N
11. Can patients be booked to return to SDEC (e.g. for follow-up or investigations) by:
    1. Acute medicine Y/N
    2. Inpatient medical wards Y/N
    3. Emergency medicine Y/N
