# Supplementary material for: Provision of medical same day emergency care services within the UK: analysis from the Society for Acute Medicine Benchmarking Audit
Source: BMJ Open. 2025 Apr 22;15(4):e094580. doi: 10.1136/bmjopen-2024-094580 (PMC12015722; doi:10.1136/bmjopen-2024-094580)
Supplement: Supplementary file 3 [file bmjopen-2024-094580.reviewer_comments.pdf]

PEER REVIEW HISTORY

BMJ Open publishes all reviews undertaken for accepted manuscripts. Reviewers are asked to complete a checklist review form and are provided with free text boxes to elaborate on their assessment. These free text comments are reproduced below.

ARTICLE DETAILS

Title (Provisional)

Provision of medical Same Day Emergency Care services within the UK: analysis from the Society for Acute Medicine Benchmarking Audit

Authors

Atkin, Catherine; Perrett, Madeleine; Cooksley, Tim; Varia, Ragit; Holland, Mark; Knight, Thomas; Subbe, Christian; Lasserson, Daniel S; Sapey, Elizabeth

---

VERSION 1 - REVIEW

**Reviewer**	**1**
**Name**	**Dean, Sue**
**Affiliation**	**United Lincolnshire Hospitals NHS Trust, Same Day Emergency Care**
**Date**	**03-Dec-2024**
**COI**	**I am a member of the Society for Acute Medicine.**

This is an excellent paper drawing on data collected through annual national audits to provide much needed evidence about same day emergency care services across the four nations of the UK. The analysis and discussion is comprehensive and the recommendations for future research are appropriate.

Page 22 line 12, repace '&' with 'and.'

Page 35 line 48, 'P' missing from Participants.

**Reviewer**	**2**
**Name**	**Barratt, Julian**
**Affiliation**	**NHS England, Centre for Advancing Practice**
**Date**	**13-Dec-2024**
**COI**	**One of my published research papers has been cited in this paper.**

I am supervising a funded PhD student undertaking funded research services.

Thanks for submitting this paper to BMJ Open - this topic area is within my area of expertise. This is an interesting and relevant paper which has been well developed. My one thought is that this paper needs some further context in the Introduction and/or Discussion section(s) to help readers more fully understand the multi-professional nature of the workforce deployed in SDEC services - particularly in relation to multi-professional advanced and consultant level practitioners who are very commonly utilised in SDEC service and often comprise a significant part of the teams in those services and re often senior decision makers in those services - this integrated feature of multi-professional advanced and consultant level practice needs to be further explicated in this paper as it is a key area of workforce development in the successful delivery of SDEC services.

**Reviewer**	**3**
**Name**	**Duffy, Richelle**
**Affiliation**	**Northumbria University, HLS**
**Date**	**16-Dec-2024**
**COI**	**None**

---

1. Some confusion with the number of sites/units. The abstract states 34,948 unplanned and 4,342 planned attendances, across 188 hospital sites.

However, the methods states patients presenting to non standard units (frailty units, specialist cancer centres, and stand-alone SDEC services) were excluded from analysis (how many units were excluded?).

Methods also states 'For analysis of survey data describing SDEC services included only in SAMBA22, participating units were stratified based on hospital size, into three groups: smaller (<400 inpatient beds, 41 hospitals), medium (400-599 inpatient beds, 50 hospitals) and larger (≥600 inpatient beds,52 hospitals)' which totals 143

Yet, the supplementary Table 1 lists: 140 in SAMBA22 structure questions and 197 units participated in any one or more collections years.

In light of the various totals please ensure when presenting data from the findings that it is clear which sample size is being used and how calculated.

2. In the abstract, p3, line 18 you state '29.8% of unplanned medical attendances received their initial medical assessment within SDEC services, with the proportion increasing over time'. Include number seen 2403 and again in p 10, line 49.

3. Please clarify which 'Selected operational standards for SDEC delivery were met in 64-91% of hospitals' you are referring to i.e. SAM/RPEC page 3, line 26.

4. 'Overall, 34.7% of medical attendances discharged without overnight admission received their medical assessment in locations other than SDEC' Page 3, line 33. Include list of other locations ED, AMU, other.

5. Page 5 para 3, consider rewording as somewhat disjointed.

6. Recommended standards page 7, line 10 clarify which standards.

7. Given the difference in consultant available discharged 'over 80% of patients assessed in SDEC the same day (72% vs 45%)' this aspect could be discussed more in discussion.

8. Be consistent with use of terms hospital, site and units as used interchangeably which causes some confusion for reader.

9. Figure legends page 22, line 43 2b add in median unit performance 85.7% and again in Figure 2 page 28, line 20.

10. Page 11, line 57 'Odds of assessment within SDEC services reduced with increasing age'. Vague term consider rephrasing. Similarly 'The likelihood of assessment in SDEC was higher in recent time' which specific time in year.

11. Table 4 is amalgamated and compared as opposed to just compared across years.

12. Use % consistently as %'s are presented in both figures and numbers.

13 Page 15, line 48 - 4.7% (309 patients) had an unplanned reattendance to ED (148 patients), AMU (23 patients) and/or SDEC (137 patients). Clarify data in this section.

patients).

VERSION 1 - AUTHOR RESPONSE

Reviewer: 1

This is an excellent paper drawing on data collected through annual national audits to provide much needed evidence about same day emergency care services across the four nations of the UK. The analysis and discussion is comprehensive and the recommendations for future research are appropriate.

- Thank you for your comments and for taking the time to review our paper. We have corrected the specific points you have mentioned.

Reviewer: 2

Thanks for submitting this paper to BMJ Open - this topic area is within my area of expertise. This is an interesting and relevant paper which has been well developed. My one thought is that this paper needs some further context in the Introduction and/or Discussion section(s) to help readers more fully understand the multi-professional nature of the workforce deployed in

SDEC services - particularly in relation to multi-professional advanced and consultant level practitioners who are very commonly utilised in SDEC service and often comprise a significant part of the teams in those services and re often senior decision makers in those services - this integrated feature of multi-professional advanced and consultant level practice needs to be further explicated in this paper as it is a key area of workforce development in the successful delivery of SDEC services.

- Thank you for your comments on the paper. We agree that your suggestions would be helpful for the reader, and have added some additional comment on the multi-professional nature of SDEC services to the introduction and to the discussion. This is focussed on the need for further understanding of optimal staffing, which we were not able to evaluate in the current dataset.

Reviewer 3:
- Thanks you for your detailed review and helpful suggestions. We have given a point by point response below.

1. Some confusion with the number of sites/units. The abstract states 34,948 unplanned and 4,342 planned attendances, across 188 hospital sites.
However, the methods states patients presenting to non standard units (frailty units, specialist cancer centres, and stand-alone SDEC services) were excluded from analysis (how many units were excluded?).

- We have added the number of each type of unit excluded to the methods section. The abstract refers to the number of included hospital sites only.

Methods also states 'For analysis of survey data describing SDEC services included only in SAMBA22, participating units were stratified based on hospital size, into three groups: smaller (<400 inpatient beds, 41 hospitals), medium (400-599 inpatient beds, 50 hospitals) and larger (≥600 inpatient beds,52 hospitals)' which totals 143
Yet, the supplementary Table 1 lists: 140 in SAMBA22 structure questions and 197 units participated in any one or more collections years.
In light of the various totals please ensure when presenting data from the findings that it is clear which sample size is being used and how calculated.

- Thank you for the suggestion. We have clarified the number of units included in each hospital size group for the SAMBA22 organisational survey comparisons in the methods, ensuring that the numbers reflect only those hospitals that responded to the SDEC service questions. We have replaced supplementary Table 1 (which previously reflected overall SAMBA participation) with a table specifically reflecting the units included in the ‘organisational survey’ section and the ‘patient level data’ section for unplanned attendances. We hope this will make it clearer for the reader.

2. In the abstract, p3, line 18 you state '29.8% of unplanned medical attendances received their initial medical assessment within SDEC services, with the proportion increasing over time'. Include number seen 2403 and again in p 10, line 49.
- We have added this.

3. Please clarify which 'Selected operational standards for SDEC delivery were met in 64-91% of hospitals' you are referring to i.e. SAM/RPEC page 3, line 26.

- We have added this.

4. 'Overall, 34.7% of medical attendances discharged without overnight admission received their medical assessment in locations other than SDEC' Page 3, line 33. Include list of other locations ED, AMU, other.
- Thank you for the suggestion, we have added this to the results section.

5. Page 5 para 3, consider rewording as somewhat disjointed.
- We have tried to amend this.

6. Recommended standards page 7, line 10 clarify which standards.
- We have clarified this.

7. Given the difference in consultant available discharged 'over 80% of patients assessed in SDEC the same day (72% vs 45%)' this aspect could be discussed more in discussion.
- Thank you for the suggestion. We do agree that this is an important finding, however within this current data, it is difficult to know whether this is reflective of a direct causal effect, or other factors that may influence the discharge rate, e.g. organisational factors or patient selection processes. We have added to the discussion to reflect this.

8. Be consistent with use of terms hospital, site and units as used interchangeably which causes some confusion for reader.
- We appreciate that this can be confusing, and have tried to be more consistent where appropriate. We feel service is likely to be the most appropriate term when referring to an SDEC, but it is not appropriate to use this throughout as we also discuss services within the SDEC service, making some paragraphs more confusing.

9. Figure legends page 22, line 43 2b add in median unit performance 85.7% and again in Figure 2 page 28, line 20.
- Thank for you for pointing out this omission – we have added it in.

10. Page 11, line 57 'Odds of assessment within SDEC services reduced with increasing age'. Vague term consider rephrasing. Similarly 'The likelihood of assessment in SDEC was higher in recent time' which specific time in year.
- We have tried to make this clearer, while avoiding repeating information from the accompanying table.

11. Table 4 is amalgamated and compared as opposed to just compared across years.
- Yes, it is across all years. We have added to the table legend to prevent any confusion.

12. Use % consistently as %'s are presented in both figures and numbers.
- We have read through to ensure percentages are presented as consistently as possible. In the text, percentages are reported as integers, except when <10% or >90%. Percentages are written out as numbers only where they start a sentence.

13 Page 15, line 48 - 4.7% (309 patients) had an unplanned reattendance to ED (148 patients), AMU (23 patients) and/or SDEC (137 patients). Clarify data in this section.
- Thank you for the suggestion. We agree the sentence is a little confusing – patients could have reattended to multiple routes, which is why it does not add up correctly and is not presented as a percentage. We have expanded on this point to try to make it clearer.

VERSION 2 - REVIEW

Reviewer 2

Name Barratt, Julian

Affiliation NHS England, Centre for Advancing Practice

Date 14-Mar-2025

COI

Thanks for undertaking the suggested revisions to the paper. In relation to the multi-professional nature of the workforce deployed in SDECs the Introduction still needs to be clearer on this and more explicitly acknowledge the contribution of advanced and consultant practitioners commonly working in SDEC services so that readers of the paper clearly understand the multi-professional nature of the advanced and consultant practitioner workforce making clinical decisions in SDECs.

For example at the end of the paragraph on page 6, "Despite the widespread adoption of SDEC as a model of care within acute services, there remains considerable variation in the provision of SDEC services between hospitals, from the specific services offered, to the multi-professional workforce used to deliver care." After "...care." at the end of the sentence it could be extended to say "...multi-professional workforce used to deliver care, whereby advanced practitioners and consultant practitioners often work in partnership with medical practitioners in those services".
